# Epidemiological and Clinical Characteristics of Neuroblastoma in Southern Iran

**Published:** 2014-07-20

**Authors:** MR Bordbar, M Tasbihi, R Kamfiroozi, S Haghpanah

**Affiliations:** 1 Hematology Research Center, Shiraz University of Medical Sciences, Shiraz, Iran; 2 Amir Oncology Hospital, Shiraz University of Medical Sciences, Shiraz, Iran

**Keywords:** Neuroblastoma, Epidemiology, Survival

## Abstract

**Background:**

Neuroblastoma is the third most common malignancy in children with a very heterogeneous feature. In this study, the epidemiological and clinical characteristics of children with neuroblastoma treated in a referral oncology hospital in Shiraz, Southern Iran, were investigated.

**Material and method:**

In this historical cohort study, the medical files of 36 children under 18 years old with neuroblastoma were reviewed, who were admitted to Amir Oncology Hospital in Shiraz, Iran from 2006 to 2013. Overall survival and event free survival (EFS) curves were demonstrated by Kaplan Meier methods. Also the effects of demographic and clinical characteristics of the patients on survival were evaluated by Cox regression model.

**Results:**

The median age of diagnosis was 30 months (age range: from 4 to144 months), with M/F ratio of 63.9%. Over 70% of the patients had stage 4 of neuroblastoma at their initial presentations. Adrenal gland comprised 72.2% of the primary tumor site. The most common presenting symptoms were gastrointestinal and constitutional symptoms. The mean overall survival and EFS were 30.75 and 20.56 months, respectively. Among the different variables analyzed, only liver metastasis had an adverse effect on EFS (p=0.025 Hazard ratios 2.83, CI: 1.14-7.02).

**Conclusion:**

This study revealed that the majority of children suffering from neuroblastoma in our center are high stage with disseminated disease at the time of detection. It also warns us about an urgent necessity for holding a re-educational program for general practitioners and pediatricians to review the warning signs of common pediatric cancers such as neuroblastoma.

## Introduction

Neuroblastoma is the third most common malignancy in the pediatric age group following leukemia and brain tumors. It is a disease with a very heterogeneous feature ranging from spontaneous regression during fetal period to disseminated metastasis at the time of diagnosis. There are a lot of risk factors which are believed to have a role in disease progression, such as age at the time of initial presentation, tumor stage, histology and ploidy of tumor, and cytogenetic aberrations including MYCN amplification, loss of heterozygosity of 1p and 11q, and 17q gain (1,2). Many large epidemiological studies have been carried out in different parts of the world focusing on the incidence, prognostic factors, and the treatment outcomes in their patients (3-8). A similar survey was conducted in Tehran, Iran to evaluate the epidemiology and outcome of their patients (9). As there was no statistics regarding the epidemiology and disease characteristics of children with neuroblastoma in Shiraz, which is the referral city for oncology patients in Southern Iran, the aim of this study is collection and analysis the epidemiological data related to pediatric patients with neuroblastoma who were admitted and treated in Amir Oncology Hospital in Shiraz, Iran.

## Materials and Methods

In this historical cohort study, all the patients are under 18 years old with confirmed diagnosis of neuroblastoma since January 2006 until April 2013, who were admitted to the pediatric oncology hospitals in Shiraz, Southern Iran. The diagnosis of neuroblastoma had been confirmed with immunohistochemistry by expert pathologists. The demographic data and the information regarding clinical presentations, sites of primary tumor, possible metastatic sites, time and site of relapse, and the patients’ outcomes were reviewed. The required information by reading their medical files were obtained, which were held in the hospital archives. International Neuroblastoma Staging System (INSS) (10) was utilized for accurate staging of the patients. As the results of N-myc amplification, DNA index and Schimada histology were not available for all of the patients, there were no in this analysis. Event-free survival (EFS) was considered as the time to first relapse or death whichever occurred first.

**Statistical analysis**

Data were analyzed by Statistical Package for the Social Sciences (SPSS, Chicago, Illinois, USA) software version 17. Survival curves were obtained by Kaplan Meier technique. Comparison of survival curves among different groups of patients was performed by Log-rank test. Cox regression model by backward LR method was performed to find out important influencing variables on survival of children with neuroblastoma. P values less than 0.05 were considered statistically significant.

## Results

Thirty six patients with diagnosis of neuroblastoma were admitted in the study period. The demographic and clinical features of the study population are summarized in Table I. Male patients comprised 66.6% of the group, and the median age of diagnosis was 30 months (age range: from 4 to144 months). Infants below the age of 18 months at the time of diagnosis constituted a quarter of admitted patients. The tumor arose most commonly in the adrenal gland (n=27, 75%), followed by cervical (n=5, 13.9%) and thoracic areas (n=4, 11.1%). Among 34 patients whose clinical and surgical data were available, 24 (70.6%) had disseminated disease (stage 4), with bone and bone marrow (BM) being the most common sites of metastasis (55.9% and 48.6% respectively), 

followed by liver (26.5%) and brain (17.6%) metastasis.Most patients presented with gastrointestinal symptoms among which abdominal mass and distension were most prevalent. 

Constitutional symptoms including anorexia, weight loss, fever, failure to thrive and pallor were the second most common presenting symptoms. Other symptoms reported frequently were abdominal pain, diarrhea, constipation, dyspnea, prolonged cough, and neurologic symptoms such as strabismus and proptosis. A small subset of patients (8.8%) showed hypertension at their initial presentations. During the 7 years of follow-up, 20 out of 34 patients (58.8%) faced disease relapse, most of them occurred in the primary site of tumor and bone (25% each), followed by BM (20%) metastasis. Moreover, 30% of relapses happened in multiple sites (25% bone and BM, 5% BM and primary site). The majority of relapses took place longer than one year from diagnosis (45%), whereas early relapse (less than 6 months) was observed in 30% of relapsed cases. Half of the patients (n=18, 50%) died due to disease progression or complications of treatment. Autologous bone marrow transplantation was performed in six patients with success rate of 33.3%. Two out of six died 5 and 18 months following transplantation due to tumor recurrence, and two of them are still under salvage chemotherapy due to regrowth of the tumor 3 and 20 months post transplantation. The other two are living free of tumor 5 and 12 months post transplantation.

**Figure 1:**
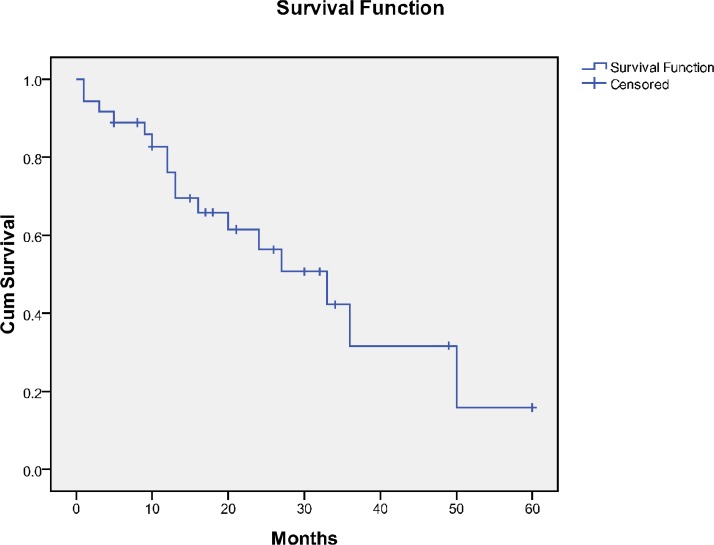
Overall survival of the children with neuroblastoma

**Figure 2:**
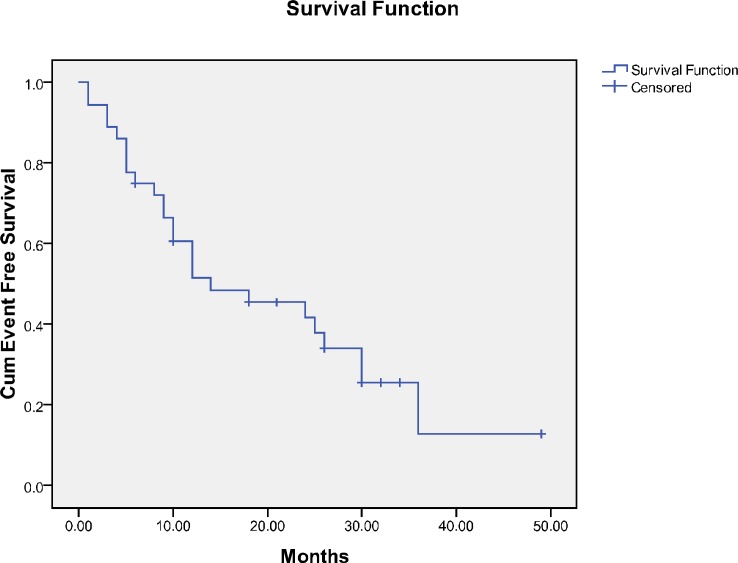
Event free survival of the children with neuroblastoma

The overall survival (OS) and EFS of the patients are demonstrated in Figure 1 and Figure 2, respectively. The median OS was 33months (95% CI 18.66-47.33), and EFS was 14 months (95% CI 1.64-26.35) (Table II). 

**Figure 3:**
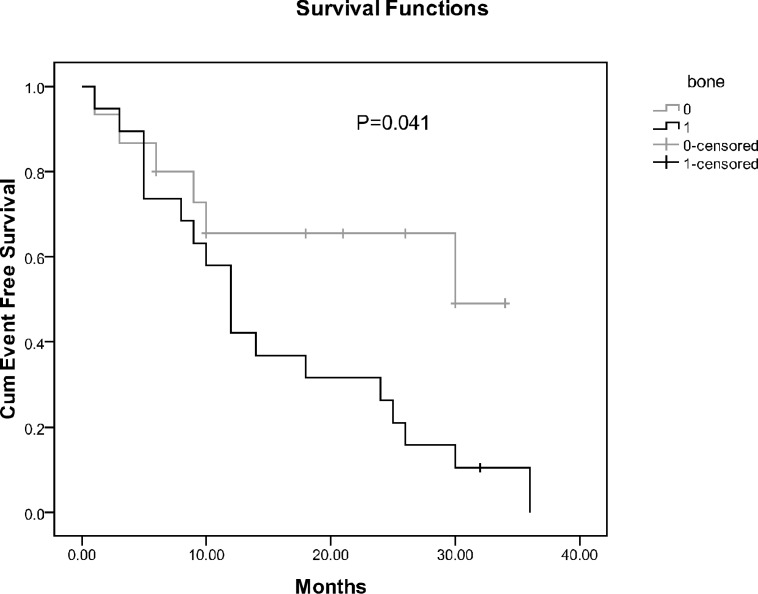
Comparison of event free survival of the children with neuroblastoma based on bone metastasis

In univariate analysis, EFS was compared between the two groups of patients regarding age at diagnosis, sex, stage of tumor, and site of metastasis as well as among different groups of patients based on primary tumor site (Table III). Only bone metastasis was shown to have a significant association with EFS (P=0.041) (Figure 3). 

**Figure 4:**
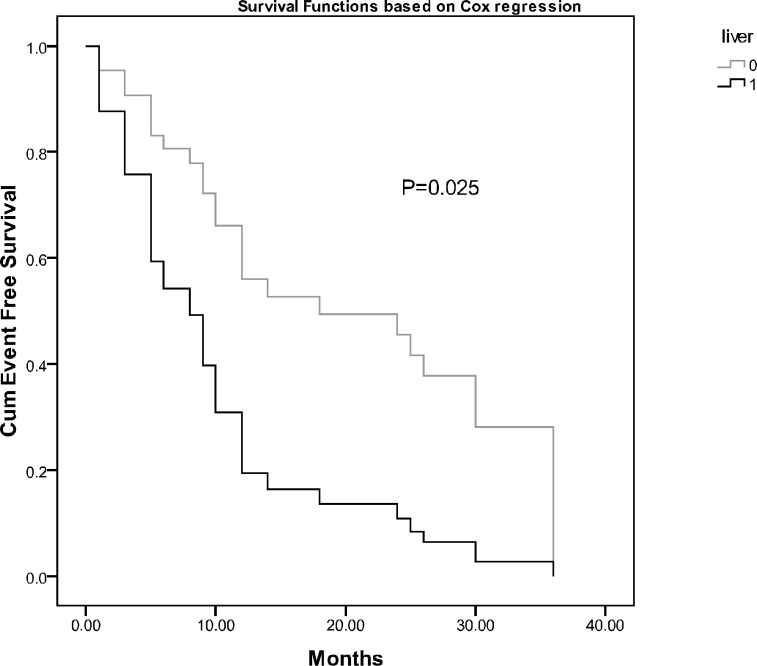
Comparison of event free survival of the children with neuroblastoma based on liver metastasis

In the next step, we conducted Cox regression analysis entering variables with significance of less than 0.25 in univariate analysis including age at diagnosis, stage, bone, bone marrow and liver metastasis. Among different variables that were supposed to have impact on EFS, only liver metastasis was shown to have prognostic significance (p=0.025 Hazard ratio 2.83, CI: 1.14-7.02) (Figure 4).

**Tabel I:** Demographic and clinical characteristics of the study population

**Variables**	**Number**	**Percent**
**Age at diagnosis (month)** **<18** **18-60** **>60**		
	9	25
	25	69.4
	2	5.6
**Sex** **Male** **Female**		
	23	63.9
	13	36.1
**Site** **Adrenal** **Thoracic** **Cervical** **Others**		
	26	72.2
	4	11.1
	5	13.9
	1	2.8
**Stage** **2** **3** **4** **4s**		
	2	5
	6	17.6
	24	70.6
	2	5.9
**Clinical presentation** **GI** **Constitutional** **Bone pain** **Others**		
	16	48.5
	10	28.5
	6	17.1
	3	8.6

**Table II:** Hormonal assessment (level of insulin, c-peptide, cortisol, GAD, ICA) and puberty stage

	**No of Events**	**Mean**	**SE**	**95% CI**	**Median**	**SE**	**95% CI**
**Overall Survival**	17 (47.2%)	30.75	4.13	22.64-38.86	33	7.31	18.66-47.33
**Event Free Survival**	25 (69.4%)	20.56	2.88	14.89-26.22	14	6.3	1.64-26.35

**Table III:** Hormonal assessment (level of insulin, c-peptide, cortisol, GAD, ICA) and puberty stage

	**Total No**	**No of events**	**Mean**	**SE**	**95% CI**	**Median**	**SE**	**95% CI**	**P value**
**Age (Month)** **<18** **>18**									
	9	3	33.98	7.05	20.15-47.8	----	----	----	0.082
	27	22	17.17	2.43	12.4-21.95	12	2.5	7.08-16.91	
**Sex** **Male** **Female**									
	23	17	18.84	2.72	13.5-24.19	12	2.39	7.3-16.69	0.871
	13	8	22.92	5.39	12.35-33.4	25	12.85	0-50.19	
**Stage** **2 and 3** **4**									
	8	3	25.25	4.79	15.85-34.64	----	----	----	0.061
	24	21	15.83	2.33	11.26-20.41	12	1.44	9.16-14.84	
**Site** **Abdominal** **Thoracic** **Cervical** **Others**									
	26	15	24.16	4.12	16.08-32.25	12	5.32	1.56-22.43	0.887
	4	4	19.25	3.63	12.12-26.37	14	6.5	1.26-26.74	
	5	5	20.6	6.11	8.6-32.58	24	16.43	0-56.2	
	1	1	12	---	12-12	12	---	----	
**Metastasis**									
**Bone** **Yes** **No**									
	19	18	15.31	2.53	10.34-20.28	12	1.43	9.18-14.81	0.041*
	15	6	23.64	3.47	16.84-30.44	30	---	----	
**Bone marrow** **Yes** **No**									
	17	12	15.15	2.88	9.49-20.8	10	1.92	6.23-13.76	0.243
	17	12	21.79	3.1	15.7-27.88	24	6.26	11.72-36.27	
**Liver** **Yes** **No**									
	9	7	14.11	4.16	5.93-22.28	10	1.41	7.22-12.77	0.121
	25	17	20.58	2.64	15.41-25.76	24	7.26	9.76-38.23	
**Brain** **Yes** **No**									
	6	5	17.5	4.78	8.11-26.88	9	9.39	0-27.4	0.728
	28	19	18.99	2.57	13.95-24.03	14	3.91	6.32-21.67	

## Discussion

In this retrospective study, the clinical and demographic characteristics of a small group of children with diagnosis of neuroblastoma were reviewed who were treated in pediatric oncology hospitals in Shiraz, Southern Iran, in a period of 7 years. The impact of different known prognostic factors on the outcome and survival of the patients were also investigated.

The demographic and clinical characteristics of our patients were quite similar to previous surveys. The disease was more prevalent in boys with M/F ratio of 1.76, comparable to most other epidemiologic studies (3,5,6,9,11,12). Most of our patients were diagnosed when they were younger than 5 years old. The mean and median ages of diagnosis were 36 and 30 months, respectively, which were similar to most previously reported studies (3,7-9,12,13). However, infants below 18 months old constituted about 25% of the patients, which is much smaller than what reported by some other investigators (3,6,14,15). Instead, the majority of our patients were in the age-range between 18 and 60 months, which is already considered as the high risk group according to the International Neuroblastoma Risk Group (INRG) task force report (16,17).Similar to other reports (3,5-9,11,18,19), adrenal gland was the most frequent site of primary disease. 

The majority of the patients had disseminated disease at the time of initial diagnosis . As the most frequently reported initial presentations were gastrointestinal and constitutional symptoms, it seems that a better understanding of the disease manifestations may lead to earlier detection and referral of new cases. It is mostly applicable to general practitioners and pediatricians whom are supposed to be in the first line of screening the children. The importance of this issue is better visualization when looking at the lower OS and EFS of our mostly high stage patients compared with similar surveys in other parts of the world (5-7,12,14,15). 

Although it has been stated in the literature that different variables such as age at the time of diagnosis, site of primary tumor, and tumor stage may have prognostic significance (2,3,5,6,9,12,15,20), none of the mentioned variables had impact on the disease outcome in our patients except for the presence of liver metastasis which led to lower EFS. Those with stage 4 disease had a more dismal prognosis compared with other stages as a whole, even though the difference was not statistically significant (P= 0.06).Similarly, infants younger than 18 months old at the time of diagnosis survived longer than older patients, but again without significant difference (P= 0.08). It can be partly explained by the small sample size of our patients in comparison to previous larger studies. Moreover, metastasis to other sites besides liver including bone marrow, and brain were not associated with higher mortality. It might be argued that liver metastasis is much more aggressive than other sites of disease dissemination, and should be treated accordingly with more intensive multimodal therapy. It is noteworthy that bone metastasis was the most common site of extra-adrenal tumor dissemination either initially or while relapses occurred. Additionally, it led to lower EFS in univariate analysis. Therefore, it seems reasonable to approach patients with bone and liver metastasis differently as they should be considered very high risk with much lower EFS if not treated appropriately.

Yalçin et al. claimed in a Cochrane systematic review that myloablative therapy may improve EFS of patients with high-risk neuroblastoma, but OS was not equally affected (21). Hence, One sixth of our patients were treated with intensive chemotherapy followed by autologous stem cell rescue. However, the results were disappointing. We could save just 2 patients out of 6 transplanted children, and the rest faced disease recurrence or died post transplantation. It is worthy of note that the time of stem cell therapy as well as the type of regimen chosen and the amount of minimal residual disease may affect the outcome of treatment (21,22).

Our study faced several limitations. In terms of cytogenetic aberrations which may influence the outcome such as amplification of MYCN proto-oncogen, DNA ploidy, allelic deletions on chromosomes 1p and 11q and gain of 17q, we had a lot of missed data, so we were unable to assess their impact on the outcome of our patients. Moreover, we had the lack of facility to consider other newly proposed genomic aberrations such as mutations in the anaplastic lymphoma kinase (ALK) gene and cyclin E1 expression (23,24). Another limitation of our study was the small sample size and nearly the short time of follow-up of the affected children. We assume that it may be one reason that some previously proposed prognostic factors such as age and disease stage did not affect the EFS of our patients. A larger multicenter survey in this area would more clearly define the epidemiologic aspects of this heterogeneous disease.

## Conclusion

In conclusion, although there has been some progress in terms of diagnosis and treatment strategies, it seems that an ongoing educational program for family physicians and pediatricians may help in earlier detection of new cases in lower stages of their diseases, which may consequently lead to better outcome and survival of the patients. Furthermore, following a more scheduled risk-adapted therapy to intensify the treatment of high-risk patients, and proper selection of eligible candidates for stem cell transplantation might improve the EFS of the patients with neuroblastoma.
